# Nutraceutical Interventions in Stunting: Advances, Challenges, and Prospects

**DOI:** 10.1002/fsn3.71910

**Published:** 2026-05-20

**Authors:** Souvia Rahimah, Trina Ekawati Tallei, Maghfirah Savitri, Chika Yamada, Hyo Jung Kim, Min Choi, Moon Nyeo Park, Youdiil Ophinni, Bonglee Kim

**Affiliations:** ^1^ Department of Food Industrial Technology, Faculty of Agroindustrial Technology Universitas Padjadjaran Bandung Indonesia; ^2^ Department of Biology, Faculty of Mathematics and Natural Sciences Sam Ratulangi University Manado Indonesia; ^3^ Department of Pathology, College of Korean Medicine Kyung Hee University Seoul Republic of Korea; ^4^ Robert Wolter Monginsidi Army Hospital Manado Indonesia; ^5^ Department of Environmental Coexistence Center for Southeast Asian Studies Kyoto Japan

**Keywords:** environmental enteric dysfunction, gut microbiome, nutraceuticals, precision nutrition, stunting

## Abstract

Childhood stunting remains a major global health challenge, reflecting the cumulative effects of inadequate nutrition, recurrent infection, and chronic intestinal dysfunction during early life. Beyond conventional micronutrient supplementation, nutraceutical interventions have emerged as complementary strategies to address the complex biological pathways underlying impaired linear growth. This review synthesizes current evidence on nutraceutical approaches to stunting, including improvements in macronutrient quality, bioactive food components, and microbiome‐targeted strategies such as probiotics, prebiotics, synbiotics, postbiotics, and microbiota‐directed foods. Evidence from clinical and preclinical studies indicates that nutraceutical effects on growth are generally modest and heterogeneous, with more consistent effects on weight gain than on height‐for‐age (HAZ). Variability in efficacy is strongly influenced by baseline nutritional status, environmental enteric dysfunction (EED), infection burden, dietary quality, and water, sanitation, and hygiene (WASH) conditions. Mechanistically, nutraceuticals may act through modulation of gut barrier integrity, inflammatory tone, microbial metabolism, and endocrine signaling pathways, particularly those involving the growth hormone–insulin‐like growth factor‐1 (GH–IGF‐1) axis. Recent microbiota‐directed food trials provide proof‐of‐concept that targeted correction of microbiome immaturity and gut dysfunction can support linear growth. Looking forward, advances in nutrigenomics, microbiome science, and epigenetics support a shift toward precision nutrition strategies that tailor interventions to biological responsiveness and context. Systems biology approaches integrating multi‐omics data, network pharmacology, and interpretable artificial intelligence are expected to refine mechanistic understanding and guide intervention design. Effective translation will require rigorous trial designs, regulatory clarity, and integration of nutraceuticals within broader stunting reduction frameworks in low‐ and middle‐income countries.

## Graphical abstract

Schematic representation of nutraceutical mechanisms in the management of stunting, illustrating an integrative pathway from pathological etiology to therapeutic outcome. (Left) Chronic malnutrition and recurrent infections contribute to gut dysbiosis and environmental enteric dysfunction (EED), leading to systemic inflammation and growth faltering. (Middle) Diverse nutraceutical interventions, including probiotics, prebiotics, essential micronutrients, amino acids, and bioactive plant extracts, target these underlying mechanisms. Key pathways include restoration of microbial equilibrium, reinforcement of the intestinal epithelial barrier, and modulation of immune responses. (Right) These physiological corrections enhance nutrient bioavailability and reactivate the growth hormone/insulin‐like growth factor‐1 (GH–IGF‐1) axis, ultimately facilitating catch‐up growth and improved cognitive development.

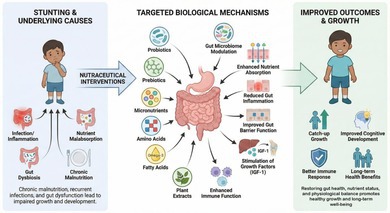

## 1 Introduction

Stunting refers to impaired linear growth in children under 5 years of age and is defined by a height‐for‐age (HAZ) value more than two standard deviations below the median of the WHO Child Growth Standards, reflecting chronic or recurrent undernutrition that often begins during the prenatal period (World Health Organization 2023). Owing to its long‐term and cumulative nature, stunting extends beyond reduced physical growth and is closely linked to lasting consequences for cognitive and neurodevelopment, compromised immune function, and an increased risk of metabolic disorders later in life, including obesity, diabetes, and hypertension (Soliman et al. 2021). When growth faltering occurs during the first 1000 days of life, from conception to 2 years of age, these adverse outcomes are particularly pronounced, as this period represents a critical window for growth and development during which the potential for catch‐up growth is limited and often incomplete (Baker 2022; Gausman et al. 2019; Leroy et al. 2020).

An estimated 149.2 million children were affected by stunting globally in 2020, underscoring its persistence as a major public health challenge and a priority within the Sustainable Development Goals (SDGs) 2030 agenda. Although global prevalence has declined over recent decades, progress has slowed in many regions, particularly in Asia, Africa, and parts of Latin America (Vaivada et al. 2020). In low‐ and middle‐income countries (LMICs), stunting rates often exceed 30%, contributing to substantial economic losses through diminished human capital, reduced workforce productivity, and private‐sector costs estimated to exceed US$135 billion annually (Akseer et al. 2022). Beyond economic impact, stunting carries profound social consequences, as affected children tend to achieve lower educational attainment and reduced lifetime earnings compared with their non‐stunted peers (Lestari et al. 2024).

In addressing the persistent burden of childhood stunting, nutraceuticals are increasingly being considered alongside established nutrition interventions (Mutumba et al. 2024; Naila et al. 2021; Ow et al. 2024; Soofi et al. 2024). The term nutraceutical, introduced by DeFelice in 1989, reflects the conceptual intersection between nutrition and pharmaceutical sciences (Kalra 2008). Despite lacking a universal definition, nutraceuticals are food‐derived bioactive compounds offering health benefits beyond basic nutrition, though inconsistent classification and limited clinical validation constrain their broader application (Nasri et al. 2014; Santini et al. 2018). Within the context of stunting, nutraceuticals are of interest for their potential to improve macronutrient and micronutrient adequacy, reduce chronic inflammation, modulate gut microbiota composition, and influence genetic and epigenetic processes involved in growth regulation (Gkiouleka et al. 2025; Jiang and Li 2025; Pauzi et al. 2025; Sharn et al. 2025; Sundjaya et al. 2024). Crucially, novel nutraceuticals can support linear growth and restore intestinal barrier function by correcting gut microbial immaturity and dysbiosis. However, despite their widespread use in chronic disease prevention, nutraceutical strategies for child growth remain underexamined compared to conventional supplementation programs.

Several systematic reviews and meta‐analyses of nutrition interventions, including single and multiple micronutrient powders (MNPs) supplementation, lipid‐based nutrient supplements (LNS), and food fortification strategies, indicate that while reductions in stunting prevalence can be achieved, consistent improvements in linear growth outcomes remain limited and highly heterogeneous (Goudet et al. 2015; Mamun et al. 2023; Matsungo et al. 2017). Key limitations include variability in trial design, contextual confounders such as poverty, poor sanitation, and recurrent infections, and a lack of precision‐targeted interventions. Moreover, well‐designed clinical trials evaluating contemporary nutraceuticals, such as bioactive peptides, selected phytochemicals, and next‐generation probiotics, as standalone or adjunctive strategies, remain limited in populations where stunting is highly prevalent (Sundjaya et al. 2024).

Furthermore, nutraceutical efficacy depends heavily on regional dietary patterns in high‐burden LMICs across Africa and Asia. Baseline diets reliant on high‐phytate staples or maize and cassava can impair nutrient bioavailability and shape the gut microbiota. Consequently, precision nutraceutical strategies must account for these distinct regional phenotypes. In addition, the application of integrated multi‐omics approaches, including genomics, epigenomics, metabolomics, and microbiome profiling, to explain inter individual responses to nutraceutical interventions is still limited in the context of childhood stunting (Escobedo‐Monge et al. 2025).

Against this backdrop, the present narrative review critically examines current evidence on nutraceutical interventions in childhood stunting, with emphasis on emerging molecular and genetic mechanisms and their relevance for precision nutrition and public health strategies in LMICs. For this narrative review, relevant literature was identified through searches of major scientific databases, including PubMed, Scopus, and Web of Science, using combinations of keywords and Boolean operators such as “nutraceuticals” AND “stunting,” “functional foods” AND “child growth,” and “dietary interventions” AND “linear growth” OR “malnutrition.” Articles were selected based on their relevance to the topic, with emphasis on recent and high‐quality studies.

## 2 Pathophysiology of Stunting

**FIGURE 1:**
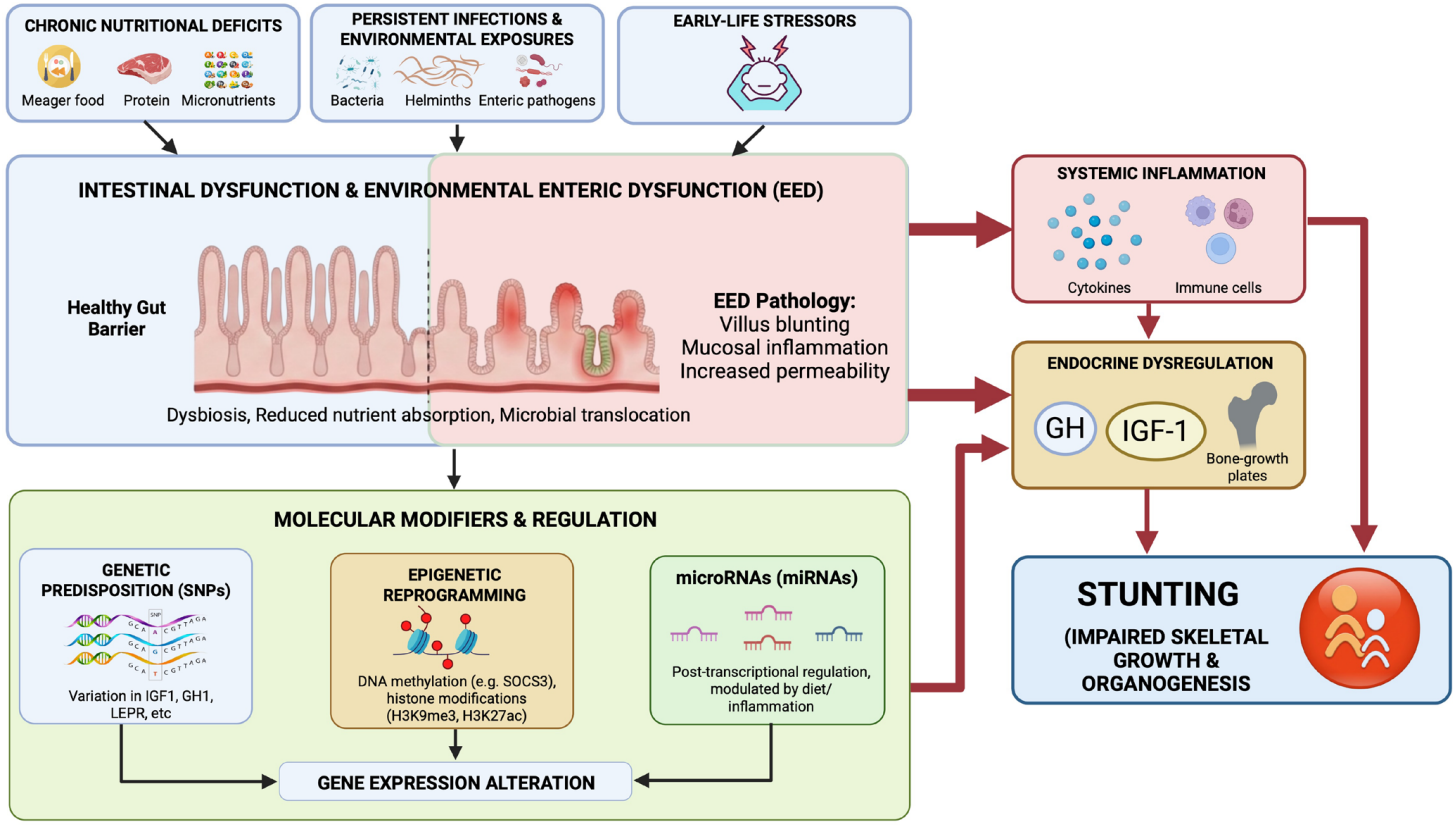
Multilevel pathophysiology of childhood stunting. Chronic nutritional deficiencies, recurrent infections, and adverse early life environmental exposures act together to disrupt intestinal function and promote EED. These processes impair nutrient absorption, sustain systemic inflammation, and interfere with signaling along the GH–IGF‐1 axis. Genetic, epigenetic, and microRNA‐mediated regulatory mechanisms further influence growth‐related pathways, culminating in impaired linear growth.

As established, the clinical phenotype of childhood stunting is the endpoint of a complex and interconnected biological process. As illustrated in Figure 1, this cycle begins at the cellular level, where insufficient intake of high‐quality protein, energy, and essential micronutrients restricts the critical substrates required for normal skeletal growth and organogenesis. Compounding this deficit, recurrent infections, particularly those caused by enteric pathogens and helminths, increase nutrient losses, elevate metabolic demands, and suppress appetite, reinforcing a cycle of malabsorption and catabolism (Gabain et al. 2023; Vonaesch et al. 2018). Central to this process is environmental enteric dysfunction (EED). As mapped in the figure, EED acts as a critical bottleneck: villus blunting, mucosal inflammation, and increased intestinal permeability severely impair nutrient absorption. The diagram highlights how this sustained physical damage creates a feedback loop of chronic immune activation that constrains linear growth, ultimately explaining why stunting often persists even alongside robust nutritional interventions (Tickell et al. 2019; Vonaesch et al. 2018).

Gut dysfunction extends systemically as microbial translocation drives low‐grade inflammation, disrupting the GH–IGF‐1 axis. This reflects an interconnected gut, brain, and growth axis where intestinal health dictates hormonal regulation and energy balance (Pauzi et al. 2025). Concurrently, microbiome alterations are heavily implicated in stunting‐associated enteropathy. Initiatives like the AFRIBIOTA project are actively characterizing these microbial patterns, though definitive signatures are still being established (Vonaesch et al. 2018).

Genetic variation in pathways that regulate growth may contribute to differences in linear growth and in responses to environmental stressors. Single nucleotide polymorphisms in genes involved in endocrine and growth regulation, including *IGF1*, *GH1*, *GHRHR*, *STAT5B*, *LEPR*, and extracellular matrix related genes such as *COMP*, have been linked to impaired growth plate function and altered GH–IGF‐1 signaling. Emerging genomic studies suggest that host genotype may influence susceptibility to stunting and responsiveness to nutritional or hormonal interventions. However, genetic testing is not routinely incorporated into clinical assessment, particularly in Asian populations, due to limited population‐specific data (Grijalva‐Avila et al. 2025; Taib and Ismail 2021). Beyond linear growth per se, *LEPR* polymorphisms have been more extensively studied in metabolic contexts and may influence growth indirectly through effects on appetite regulation, energy balance, and endocrine signaling. For example, interactions between *LEPR* variants and microRNAs have been explored in hypertension risk, illustrating how genetic variation in growth‐regulatory pathways can intersect with post‐transcriptional control mechanisms (Kim and Hong 2023). Similarly, variable responses to growth hormone therapy in children born small for gestational age point to underlying genetic or epigenetic factors that shape individual growth trajectories (Tian et al. 2024).

Early life environmental factors, including nutrition and infection, shape growth trajectories through epigenetic modifications such as DNA methylation and histone changes (Breton et al. 2021; Tian and Marsit 2018). Specifically, stunted Bangladeshi children exhibited higher global DNA methylation linked to reduced macronutrient and zinc intake (Iqbal et al. 2019). Furthermore, longitudinal studies connect stunting to early immune activation via increased H3K27 acetylation in infants (Kupkova et al. 2021), alongside elevated repressive H3K9 trimethylation at growth and metabolic genes by 1 year of age (Kupkova et al. 2021).

At the gene‐specific level, epigenome‐wide studies in LMICs cohorts have identified DNA methylation at the *SOCS3* locus as a strong predictor of child height, independent of underlying genetic variation. Experimental and causal analyses suggest that *SOCS3* methylation functions as a regulatory link between inflammatory signaling and growth control (Issarapu et al. 2022). Complementing these findings, the UKRI Action Against Stunting Hub has initiated longitudinal epigenetic studies in India, Indonesia, and Senegal to map early life DNA methylation patterns and examine their associations with stunting risk and responsiveness to nutritional interventions. Findings from these studies are expected to clarify how epigenetic regulation contributes to growth outcomes, although results are not yet available (Ramsteijn et al. 2024).

In addition to DNA methylation and histone modifications, microRNAs (miRNAs) represent another layer of epigenetic regulation relevant to growth and development. MiRNAs represent a critical layer of epigenetic regulation, modulating gene expression post‐transcriptionally to influence linear growth, inflammation, and nutrient metabolism (Oliveto et al. 2025). Pathophysiologically, inflammation‐associated miRNAs disrupt growth hormone and insulin‐like growth factor (GH–IGF‐1) signaling, thereby impairing linear growth (Cirillo et al. 2018). Consistent with this mechanism, pediatric intestinal studies have linked miRNAs such as miR‐21 and miR‐122 to markers of intestinal permeability and inflammation in children living in high pathogen‐burden environments (Rashid et al. 2021).

Furthermore, early life nutrition heavily influences these pathways. Maternal malnutrition and specific dietary factors dynamically modulate offspring miRNA profiles, altering developmental trajectories and hormone‐related signaling (Ağagündüz et al. 2025; Martino et al. 2024; Sasso et al. 2024; Zeng et al. 2022). While genetic polymorphisms introduce further miRNA regulatory complexity (Cao et al. 2024; Khan et al. 2024), their direct relevance to childhood stunting remains to be established.

## 3 Nutraceuticals Interventions: Targeting the Biology of Stunting

To overcome the biological drivers of stunting, interventions must move beyond basic nutrition to target enteric dysfunction and systemic inflammation. Nutraceuticals, such as phytochemicals and biotics, offer a means to repair the intestinal barrier and resolve inflammatory burdens. These targeted therapies aim to transition the host from an inflammation‐driven catabolic state to an anabolic state, restoring the hormonal regulation necessary for linear growth. The following sections evaluate the efficacy and mechanisms of these interventions.

### 3.1 Macronutrient‐Based Nutraceuticals

**FIGURE 2:**
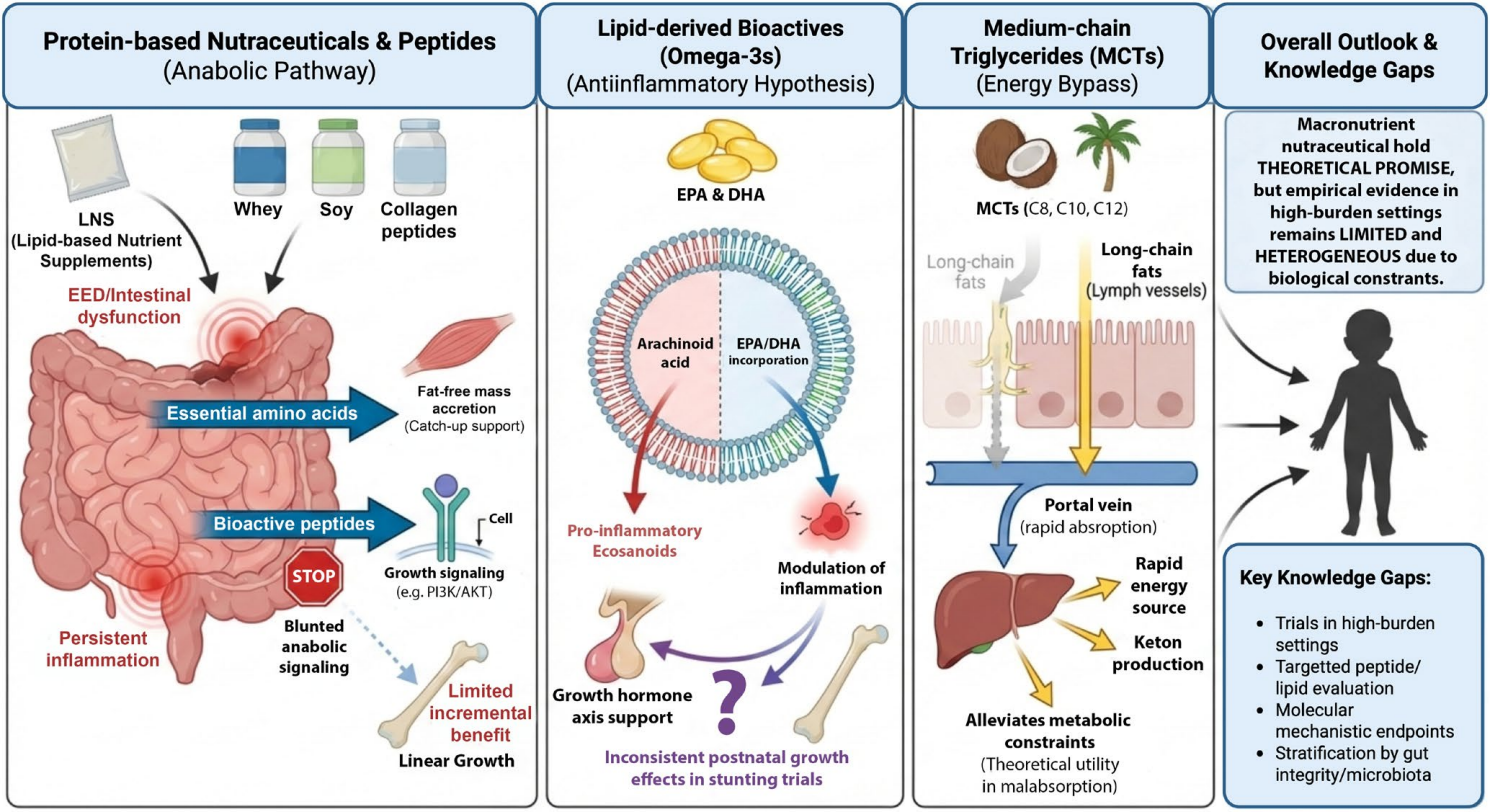
Macronutrient‐based nutraceuticals in childhood stunting: A conceptual overview summarizing proposed mechanisms, evidence strength, and major knowledge gaps for protein‐based nutraceuticals, omega‐3 fatty acids, and medium‐chain triglycerides (MCTs) in relation to linear growth in stunting‐prone settings.

Macronutrient‐focused nutraceuticals are designed to provide high‐quality nutritional substrates, particularly proteins and bioactive lipids, that support not only basal growth requirements but also regulate metabolic, endocrine, and intracellular signaling pathways critical for linear growth. However, as illustrated in Figure 2, such interventions may partially mitigate inadequate dietary intake, impaired absorption, and inflammation‐driven catabolic states that constrain growth trajectories in stunting‐prone settings (Braun et al. 2016; Delplanque et al. 2015; Okai‐Mensah et al. 2025).

Protein‐based nutraceuticals, including whey and bioactive peptides, are often incorporated into LNS. Evidence from randomized trials in children aged 12–59 months indicates that the addition of dairy components to LNS does not confer additional benefits for linear growth or body composition compared with standard non‐dairy LNS formulations. Nevertheless, LNS supports linear catch‐up growth and fat‐free mass accretion, improving growth quality compared to untreated stunted children who often accrue fat mass instead (Mbabazi et al. 2023). Although LNS reduces systemic inflammation, the MAGNUS trial found that enrichment with milk protein or whey permeate provided no additional linear growth benefits (Mutumba et al. 2025). These findings suggest that protein enrichment provides limited additional growth benefits for children already receiving LNS. This likely stems from biological constraints, such as intestinal dysfunction and inflammation, which impair nutrient absorption and anabolic signaling. These disturbances establish a vicious cycle where malabsorption and gut damage perpetuate undernutrition in stunted toddlers (Amimo et al. 2025).

While LNS have been extensively evaluated in large‐scale pediatric randomized controlled trials (RCTs), demonstrating tangible benefits for catch‐up growth, other macronutrient therapies remain largely theoretical. For instance, although in vitro and animal models show that isolated bioactive peptides (e.g., soy‐derived sequences) can modulate PI3K/Akt signaling, direct empirical evidence from clinical trials evaluating these peptides in stunted children is currently lacking (Liu et al. 2024; Yan et al. 2025). However, to date there is limited direct evidence of these individual peptides being evaluated as nutraceuticals for childhood stunting, representing a promising frontier for future research.

Lipid‐derived bioactives, particularly long‐chain omega‐3 polyunsaturated fatty acids (PUFAs), are recognized for their roles in neurodevelopment, immune modulation, and anti‐inflammatory signaling, and have been hypothesized to support growth through effects on membrane dynamics and endocrine pathways. However, evidence from supplementation studies in pregnant women, infants, and young children indicates that, aside from modest increases in birth weight and gestational duration, omega‐3 fatty acid supplementation does not consistently improve postnatal growth or other maternal and infant health outcomes, with reported effects varying across study designs and endpoints (Newberry et al. 2016).

In pediatric populations with short stature, though not in stunting‐endemic settings, omega‐3 supplementation has been associated with modest improvements in height standard deviation scores independent of growth hormone therapy; for example, a small intervention study (*n* = 34) reported an increase in height SDS from −2.2 to −2.0 over approximately 245 days. In contrast, randomized trials of docosahexaenoic acid (DHA) and other omega‐3 long‐chain PUFAs administered during pregnancy, lactation, or infancy show inconsistent benefits, with variable effects on visual acuity and limited neurodevelopmental gains, but no consistent or sustained impact on postnatal linear growth (Campoy et al. 2012). Evidence from cognition‐focused trials indicates that the biological efficacy of omega‐3 supplementation may depend on achieving sufficient exposure, commonly reflected by daily intakes of ≥ 450 mg DHA + eicosapentaenoic acid (EPA) or an omega‐3 index above ~6% (van der Wurff et al. 2020). However, whether similar dose thresholds or biomarker targets are required to influence linear growth or childhood stunting remains unknown, as growth‐related outcomes were not assessed in these studies. Interpretation of the broader growth literature is further limited by small sample sizes, incomplete blinding, and the underrepresentation of children from high‐burden LMICs.

Systematic reviews of omega‐3 supplementation show inconsistent neurodevelopmental outcomes but suggest potential benefits for physical growth and short‐term cognition (Sherzai et al. 2022; Zulhazman et al. 2025). In the context of stunting, where EED‐induced inflammation disrupts growth hormone signaling (Kadia et al. 2024), omega‐3 s may be beneficial through anti‐inflammatory pathways. Specifically, incorporating EPA into cell membranes dampens pro‐inflammatory signaling by reducing arachidonic acid‐derived eicosanoid synthesis (Banaszak et al. 2024).

Medium‐chain triglycerides (MCTs), composed primarily of saturated fatty acids ranging from caprylic (C8:0) to capric (C10:0), and in some formulations lauric acid (C12:0), are rapidly absorbed and bypass conventional intestinal lipid transport, entering the portal circulation for immediate metabolism (Cao et al. 2025). In malabsorptive conditions like EED, MCTs provide an accessible energy source that helps alleviate metabolic growth constraints (Zulfakar et al. 2024). While established in therapeutic formulas for pediatric malnutrition, their role as nutraceutical adjuncts for stunting remains under‐investigated. Due to their rapid oxidation and limited fat storage (Shcherbakova et al. 2022), MCTs could effectively complement protein and micronutrient interventions.

While macronutrient‐based nutraceuticals are theoretically promising, empirical evidence for dairy‐enriched LNS and omega‐3 s remains inconsistent and lacks definitive linear growth benefits. Significant research gaps persist, including a shortage of trials in high‐burden settings and a lack of molecular endpoints such as IGF‐axis activity or epigenetic shifts. Furthermore, few studies account for host factors like gut integrity and microbiota composition. Closing these gaps requires integrative research that combines nutraceutical interventions with multi‐omic biomarkers to define efficacy and mechanisms in stunting‐endemic populations.

### 3.2 Micronutrient‐Focused Nutraceuticals

The critical role of micronutrients in supporting cellular proliferation and immune function is well‐established through fundamental in vitro and biochemical research (Pecora et al. 2020). The inadequate intake or low bioavailability of key elements, including zinc, iron, iodine, and vitamins A, D, and folate, directly disrupts growth pathways and increases vulnerability to chronic inflammation. Consequently, micronutrient‐focused nutraceuticals utilize targeted supplementation, point‐of‐use (or home) fortification with MNPs, and multiple micronutrient (MMN) formulations to address these concurrent deficiencies (Mulyani et al. 2025). However, when translated to clinical settings, evidence from pediatric RCTs and meta‐analyses reveals substantial heterogeneity. For example, while the theoretical need for iron is clear, large‐scale human trials report inconsistent effects on linear growth, particularly in populations without overt anemia.

Zinc is an essential micronutrient for growth, immune competence, and neurodevelopment, with deficiency contributing to growth retardation, increased infection risk, and impaired cognitive and motor function, particularly in vulnerable populations (Majumdar et al. 2025). A meta‐analysis of eight studies (*n* = 1586) demonstrated that zinc supplementation significantly improved height, weight, and HAZ scores, with no significant dose–duration relationship observed in anthropometric outcomes (Monfared et al. 2023). Zinc supplementation significantly enhances linear growth and weight gain in stunted infants, often restoring growth rates to levels comparable with non‐stunted peers by improving appetite and reducing infection‐related morbidity (Umeta et al. 2000). However, trial results remain heterogeneous, likely due to variations in baseline zinc status, nutrient interactions, and confounding factors such as enteric dysfunction (Long et al. 2019). A dynamic kinetic model suggests that smaller, more frequent zinc dosing (5–10 mg/d) may outperform larger bolus regimens in supporting growth, particularly in zinc‐deficient, stunted infants (Wastney et al. 2018).

Iron deficiency is a leading cause of anemia and can severely impair neurodevelopment. Beyond its role in oxygen transport, prolonged deficiency in early life constrains growth by reducing tissue oxygenation and increasing morbidity, with some cognitive effects remaining irreversible even after correction (Ibrahim et al. 2017; Leung et al. 2023). Early evidence from anemic preschool children in Indonesia showed that 2 months of iron supplementation (30 mg iron plus 20 mg vitamin C) significantly improved linear growth and reduced stunting progression (Angeles et al. 1993). However, subsequent larger trials have reported inconsistent effects on linear growth, particularly when iron is administered alone or in populations without overt anemia (Rahman et al. 1999). Iron supplementation may also increase oxidative stress and alter host–pathogen interactions under inflammatory or infectious conditions, suggesting that background health status modulates growth responses to iron therapy (Obeagu 2025; Raffaeli et al. 2020).

Iodine is indispensable for thyroid hormone synthesis, which regulates basal metabolic activity and plays a central role in somatic growth and neurodevelopment, particularly during the first 1000 days of life (Velasco et al. 2018). While iodine deficiency is a well‐recognized cause of cognitive impairment and cretinism, evidence that iodine supplementation consistently improves linear growth in stunted children remains limited, with measurable effects largely confined to settings of severe deficiency. As a result, the role of iodine within nutraceutical strategies for stunting is largely inferential and underscores the need for targeted intervention trials in iodine‐deficient populations, supported by epidemiological findings identifying iodine deficiency disorders as a predictor of stunting among primary school children in the Aseer region of southwestern Saudi Arabia (Abbag et al. 2021).

Vitamin A is vital for immunity and epithelial integrity, yet supplementation alone does not reliably improve linear growth, typically yielding only modest gains in weight (Gürbüz and Aktaç 2022; Imdad et al. 2017; Peng et al. 2025). Similarly, while vitamin D is essential for bone metabolism and immune modulation, clinical evidence linking it to meaningful catch‐up growth in LMICs remains limited and inconsistent. These findings underscore the need for multi‐nutrient, context‐sensitive strategies rather than isolated vitamin interventions. Long‐term weekly vitamin D_3_ supplementation improves serum 25 (OH)D but does not affect growth, body composition, or puberty in school‐aged children (Ganmaa et al. 2022). In Afghan infants, vitamin D_3_ trials showed only non‐significant linear growth effects, with minor benefits limited to subgroups with high calcium intake (Crowe et al. 2020). Consequently, a Cochrane review found that vitamin D results in little to no improvement in stunting outcomes (Huey et al. 2020). These results suggest that vitamin D alone is insufficient without adequate calcium and proper bone mineralization regulation. Vitamin K_2_ may assist by activating osteocalcin to regulate skeletal calcium deposition (Aaseth et al. 2024; Al‐Suhaimi and Al‐Jafary 2019), though its specific role in linear growth requires further study. Effective vitamin D‐based nutraceutical strategies likely require complementary nutrients and trials that account for baseline deficiencies and infection burden.

Folate (vitamin B_9_), together with vitamin B_12_, is central to one‐carbon metabolism, supporting the DNA synthesis and methylation required for cell division and tissue growth (Lyon et al. 2020). While inadequacy can theoretically constrain growth by limiting cellular proliferation, clinical evidence remains modest. A large factorial RCT in North Indian children found that folate and B_12_ supplementation primarily improved weight, with HAZ gains restricted to undernourished subgroups receiving vitamin B_12_ (Strand et al. 2015). Longer‐term follow‐up of the same cohort found no overall effect of folic acid and only a small, non‐significant average effect of vitamin B_12_ on height, with clearer benefits limited to children with low baseline cobalamin status (Taneja et al. 2022).

Beyond individual nutrients, micronutrient powders (MNPs) offer a practical point‐of‐use fortification platform. A systematic review found MNPs as effective as iron drops for anemia, with better acceptability, but noted they only improve child growth when combined with caloric energy (Dewey et al. 2009). Trial results for linear growth remain mixed. In full‐term, low‐birth‐weight infants, a 22‐nutrient MNP delivered with health education significantly reduced stunting odds at 12 months (OR ≈ 0.35) compared to education alone (Shafique et al. 2016). In the Gaza Strip, providing three MNP sachets weekly alongside national programs over 12 months led to a 1% stunting prevalence versus 11% in controls (Albelbeisi et al. 2020). Conversely, a study in Burkina Faso involving children aged 6–23 months showed only modest length‐for‐age (LAZ) gains when MNPs were paired with behavior change education (Lanou et al. 2019). While MNPs consistently improve anemia and micronutrient status, their impact on linear growth across diverse settings remains inconsistent.

**FIGURE 3:**
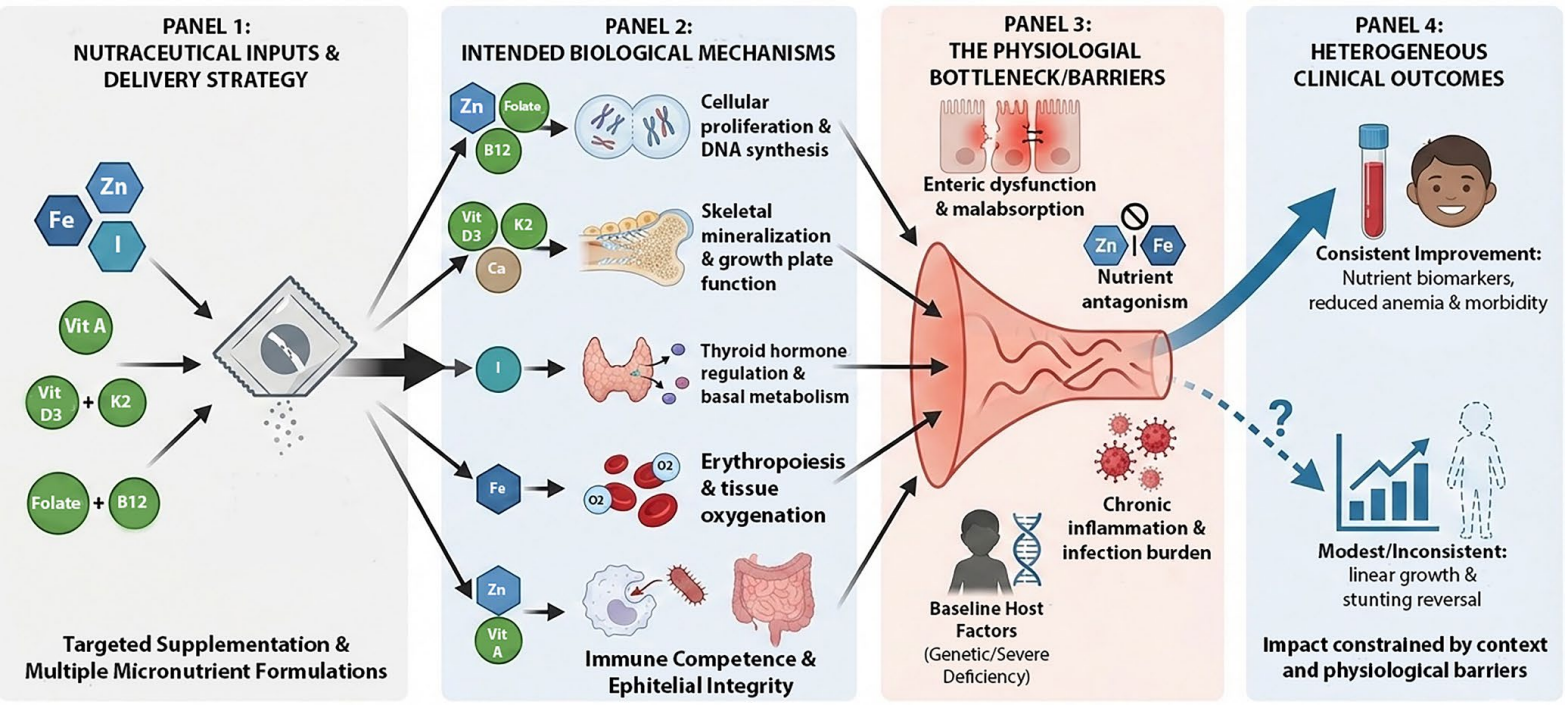
Mechanistic pathways and physiological barriers modulating the efficacy of micronutrient nutraceuticals on linear growth. Panel 1 depicts nutraceutical inputs and delivery strategies, including point‐of‐use fortification (e.g., micronutrient powders/sprinkles) and targeted supplementation with single or multiple micronutrients. Panel 2 illustrates intended biological mechanisms, whereby micronutrients (e.g., zinc, iron, iodine, vitamins A and D, folate/B_12_) support cellular proliferation, skeletal mineralization and growth plate function, thyroid hormone synthesis and basal metabolism, erythropoiesis, and immune competence. Panel 3 highlights key physiological bottlenecks and barriers that limit growth responses, such as enteric dysfunction and malabsorption, nutrient antagonism, chronic inflammation or infection, and baseline host factors including deficiency status and genetics. Panel 4 summarizes heterogeneous clinical outcomes, showing consistent improvements in nutrient biomarkers and morbidity reduction, but only modest, inconsistent, and context‐dependent effects on linear growth and stunting reversal across populations.

As summarized in Figure 3, which brings together mechanistic pathways, physiological limitations, and clinical outcomes, existing evidence supports the biological rationale for micronutrient‐focused nutraceuticals and their consistent benefits for improving micronutrient status and reducing morbidity. In contrast, their effects on linear growth and stunting reversal remain limited and strongly influenced by context. Several factors contribute to this gap, including impaired nutrient absorption due to enteric dysfunction, antagonistic interactions among micronutrients such as zinc and iron, inadequate stratification by baseline nutritional status or host characteristics, and the limited incorporation of molecular or endocrine intermediates, including epigenetic markers or insulin‐like growth factor signaling, in clinical studies. Future work should therefore emphasize rational nutrient combinations, optimized dosing strategies, and integration with interventions that target gut health to improve growth outcomes in populations with a high stunting burden.

### 3.3 Phytochemicals and Bioactive Compounds

**FIGURE 4:**
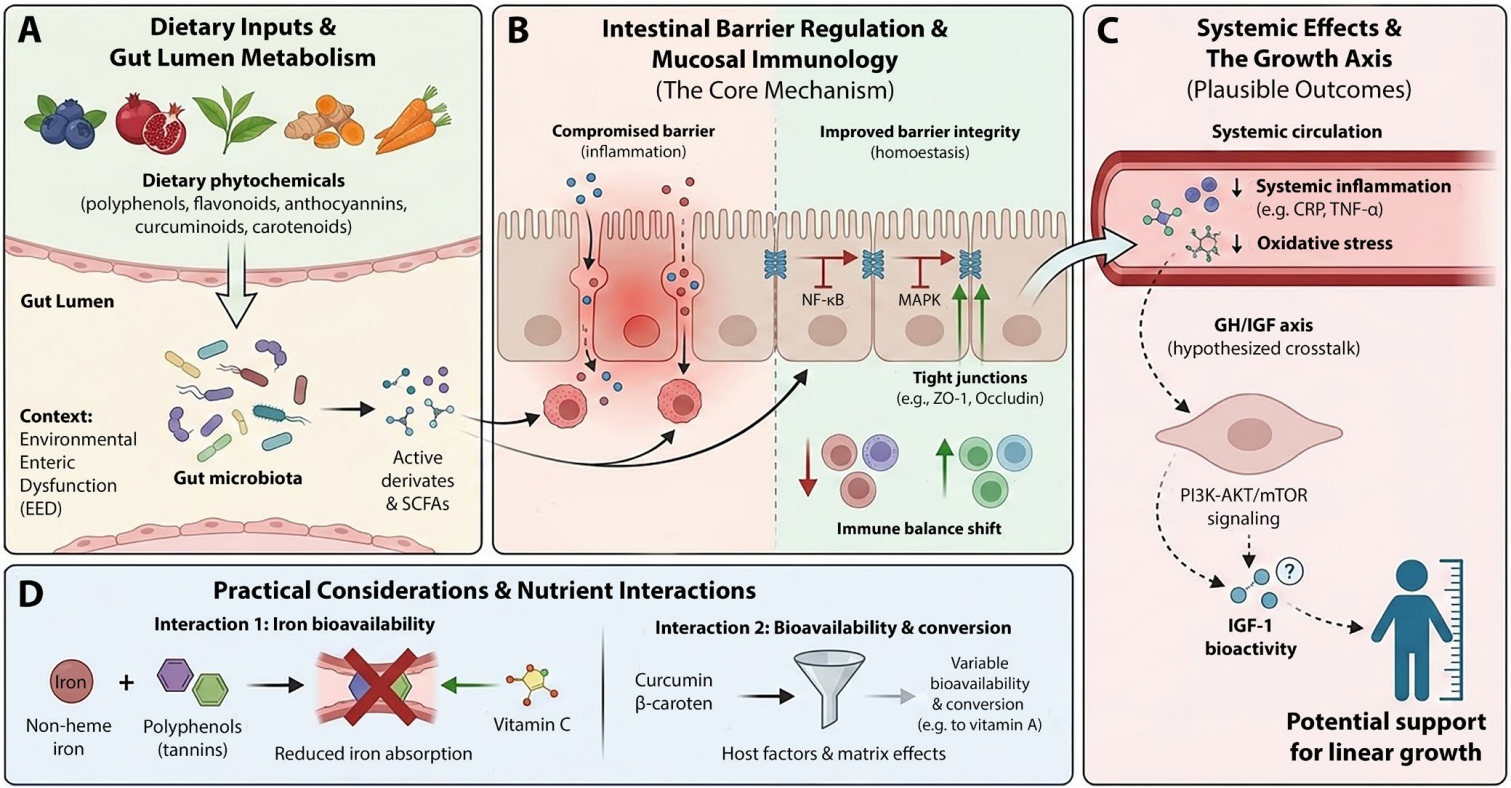
Proposed mechanisms and practical considerations for phytochemicals in supporting linear growth. (A) Dietary phytochemicals are metabolized by the gut microbiota into active derivatives and SCFAs, particularly relevant in EED. (B) These metabolites suppress inflammatory signaling (NF‐κB, MAPK) and shift immune balance (↓Th17/↑Treg), leading to the upregulation of tight junction proteins (ZO‐1, Occludin) and barrier restoration. (C) Reduced systemic inflammation and oxidative stress provide a plausible basis for crosstalk with the GH/IGF axis, potentially modulating PI3K–AKT/mTOR signaling to support growth (dashed lines indicate unconfirmed pathways). (D) Practical challenges include the inhibition of non‐heme iron absorption by tannins (mitigated by vitamin C) and variable bioavailability or conversion of lipophilic compounds like curcumin and β‐carotene.

Phytochemicals represent a distinct class of gut‐active nutraceuticals with mechanistic and practical relevance to linear growth. Dietary phytochemicals are increasingly examined as adjuncts to conventional nutrition because they can attenuate intestinal inflammation, reinforce epithelial barrier integrity, modulate homeostatic equilibrium, and secondarily influence endocrine cues relevant to growth (Nemzer et al. 2025; Solís‐S et al. 2017). Preclinical evidence heavily supports these anti‐inflammatory properties. Numerous in vitro assays and murine models demonstrate these effects, and Figure 4 details the specific mechanisms by which subclasses like flavonoids and curcumin downregulate pro‐inflammatory cytokines, repair intestinal barriers, and are metabolized by the microbiota to shift Th17/Treg immune dynamics (Fu et al. 2025; Kelepouri et al. 2018; Sun et al. 2024). Nevertheless, a critical gap remains, as robust RCTs have not yet validated whether these mechanistic, preclinical benefits translate into measurable linear growth improvements, such as increased HAZ or LAZ z‐scores, in stunted children.

In humans, polyphenol‐rich foods enrich short‐chain fatty acid (SCFA)‐producing microbiota, improving epithelial homeostasis and lowering systemic inflammation. Coupled with direct antioxidant effects, these mechanisms suggest polyphenols can reinforce intestinal barrier integrity and mitigate mucosal inflammation. These effects are essential prerequisites for linear growth when combined with adequate nutrition (Nemzer et al. 2025; Wang et al. 2022).

Although direct pediatric trials are lacking, experimental evidence suggests flavonoids may intersect with GH–IGF‐1 axis. In vitro and disease models demonstrate that flavonoids modulate PI3K/AKT/mTOR pathways, establishing biological plausibility for crosstalk with IGF signaling (Shanak et al. 2022; Stachelska et al. 2025). This mechanism strongly parallels metabolic disease research, where natural compounds regulate insulin signaling and PI3K/AKT‐dependent cellular metabolism, providing a conceptual basis for exploring similar phytochemical effects on endocrine growth pathways (Ansari et al. 2024; Savova et al. 2023).

Small human studies indicate anthocyanin interventions alter cyclic glycine‐proline (cGP), a metabolite influencing IGF‐1 bioactivity, without changing circulating IGF‐1 or IGFBP‐3 (Fan et al. 2018), and an open‐label follow‐up suggests cGP supplementation may normalize IGF‐1–mediated vascular remodeling (Chen et al. 2025). Broadly, flavonoids exert antioxidant, anti‐inflammatory, and microbiota‐modulating effects rather than directly modulating IGF in children. Nevertheless, individual compounds display diverse biological actions. For instance, quercetin impacts IGF‐related tissue repair, while epigallocatechin gallate (EGCG) demonstrates cognitive benefits in children with Down syndrome (Melrose 2023). Conversely, pediatric zinc trials in rural Laos reported no significant changes in IGF‐1 or IGFBP‐3, although baseline IGF status modified subgroup growth responses (Barffour et al. 2023). Consequently, future trials studying bioactive compounds like flavonoids must define IGF outcomes a priori. Ultimately, the GH–IGF‐1 axis remains a plausible but unconfirmed target for flavonoid interventions, necessitating well‐designed trials in high‐burden settings that measure IGF‐1, IGFBP‐3, and downstream PI3K/AKT signaling.

Curcuminoids are extensively researched phytochemicals with potent anti‐inflammatory and antioxidant properties. Comprehensive clinical meta‐analyses demonstrate that curcumin supplementation significantly lowers key inflammatory markers (CRP, TNF‐α, IL‐6) and oxidative stress (MDA) while boosting antioxidant enzyme activity (SOD, GPx, CAT). Additionally, curcumin improves endothelial function, evidenced by reduced pulse wave velocity and enhanced flow‐mediated dilation. However, total antioxidant capacity does not consistently increase, indicating that its positive effects on oxidative and vascular biomarkers are not strictly uniform across all measures (Kavyani et al. 2024; Zhang et al. 2022).

Evidence from pediatric cohorts with inflammatory bowel disease further supports its clinical relevance: a multicenter study in children with ulcerative colitis found that curcumin, in combination with Qing Dai, improved disease activity and was well tolerated as an adjunct to standard therapy (Nachum et al. 2024). Mechanistically, curcumin reduces murine colitis severity by increasing anti‐inflammatory IL‐10, suppressing pro‐inflammatory cytokines (IL‐1β, IL‐6, TNF‐α), and reinforcing epithelial barriers primarily via gut microbiota modulation (Yi et al. 2025).

Although not directly studying stunting, the shared mucosal inflammation and barrier dysfunction between ulcerative colitis and EED indicate curcuminoids could serve as adjuncts in future growth interventions. Realizing this potential necessitates enhanced bioavailability, strict pediatric safety assessments, and well‐designed trials evaluating mechanistic and clinical growth outcomes (Heidari et al. 2022; Mirjalili et al. 2024).

Carotenoids (e.g., β‐carotene, lutein, zeaxanthin) are C40 pigments with potent antioxidant, anti‐inflammatory, and neuroprotective properties (Shanaida et al. 2025; Tallei et al. 2023). Recent prenatal studies indicate that lutein and zeaxanthin supplementation during pregnancy improves maternal–infant carotenoid status and ocular biomarkers, with implications for perinatal antioxidant protection and early visual development (Addo et al. 2024; Arunkumar et al. 2023). While direct impacts on postnatal linear growth remain unproven, reducing early developmental oxidative stress may indirectly support growth trajectories in high‐burden settings.

Conversely, β‐carotene's nutritional value depends heavily on its bioavailability and conversion to vitamin A. This conversion is highly variable, influenced by the food matrix, dietary composition, and host factors like baseline vitamin A status, coexisting deficiencies, gut health, infections, inflammation, and genetic polymorphisms (Haskell 2012). These variables cause significant interindividual and population‐level heterogeneity, demanding careful consideration when deploying carotenoid‐rich nutraceuticals in LMIC contexts.

Incorporating polyphenol‐based ingredients into stunting‐focused programs requires two practical considerations. First, tannins and other polyphenols inhibit non‐heme iron absorption, a major concern in iron‐deficient populations (He and Chen 2024). Separating polyphenol‐rich items from iron‐dense meals or co‐administering ascorbic acid can minimize this inhibition (Piskin et al. 2022). Second, the low solubility of curcumin (Górnicka et al. 2023) and the variable efficiency of carotenoid conversion to retinol (Adamantidi et al. 2025) necessitate biomarker‐informed dosing and advanced formulations. Technologies like nanoencapsulation or lipid matrices are essential to protect sensitive bioactives from metabolic degradation and improve stability in the compromised pediatric gut.

Recent literature supports the biological plausibility, though not yet definitive clinical efficacy, of phytochemicals as adjuncts to stunting prevention/treatment (Budiono and Has 2025; Yarmaliza et al. 2024). Future RCTs in stunting‐endemic pediatric populations must evaluate mechanistic outcomes, including intestinal barrier permeability, inflammatory mediators, short‐chain fatty acids, and IGF axis indicators. Addressing these evidence gaps and accounting for nutrient interactions will critically inform next‐generation nutraceutical research integrated within broader nutrition and infection control frameworks.

### 3.4 Probiotics, Prebiotics, Synbiotics, and Postbiotics

#### 3.4.1 Gut Microbiome Modulation

Linear growth faltering is increasingly linked to gut ecosystem dysfunction, characterized by the convergence of microbiome immaturity or dysbiosis, impaired intestinal barrier integrity with microbial translocation, and chronic low‐grade inflammation consistent with EED. Within this framework, microbiome‐targeted nutraceutical interventions are proposed to redirect nutrient utilization away from inflammation‐driven catabolism toward anabolic growth processes by restoring gut barrier function and host–microbe metabolic and immunological signaling (Mostafa, Hibberd, et al. 2024; Otiti et al. 2025; Pauzi et al. 2025).

Probiotics, defined as live microorganisms conferring health benefits, may modulate growth‐relevant biological processes in a strain‐dependent manner. These effects include enhancement of intestinal barrier integrity through tight‐junction regulation and mucus layer support, thereby limiting endotoxin translocation and systemic inflammatory burden. Probiotics may also recalibrate immune tone by attenuating pro‐inflammatory cytokine signaling that suppresses GH–IGF‐1 axis. In addition, modulation of microbial metabolic activity, particularly increased production of short‐chain fatty acids such as acetate and butyrate, supports colonocyte energy metabolism, promotes epithelial repair, and exerts anti‐inflammatory effects via G‐protein–coupled receptor and histone deacetylase–related signaling pathways (Otiti et al. 2025; Pauzi et al. 2025).

While probiotics act primarily through the direct introduction of functional microbial strains, complementary benefits can also be achieved by selectively promoting the growth and activity of endogenous beneficial microbes through prebiotic substrates. Prebiotics, selectively utilized substrates that confer host benefits, traditionally include galacto‐ and fructo‐oligosaccharides (GOS/FOS) and, more recently, industrially produced human milk oligosaccharides (HMOs). Their primary mode of action involves selective enrichment of beneficial microbial taxa, most notably *Bifidobacterium* species during early life, leading to increased short‐chain fatty acid production and enhanced ecological stability of the gut microbiome. This mechanism is particularly relevant when early HMO exposure is reduced, such as in partial or absent breastfeeding, as disruption of “infant‐type” bifidobacterial networks may compromise nutrient utilization efficiency and immune calibration during critical growth periods (Kebbe et al. 2025; Nuzhat et al. 2023).

Building on this principle, synbiotics seek to combine both approaches within a single, coordinated intervention. Synbiotics, which combine a probiotic with a complementary prebiotic, are conceptually advantageous when the selected substrate is specifically designed to support the growth and metabolic activity of the administered strain or a targeted microbial guild. However, empirical evidence indicates that synbiotic efficacy is highly context‐dependent, requiring careful alignment among probiotic strain characteristics, prebiotic substrate specificity, background dietary patterns, and prevailing pathogen pressure, rather than assuming uniform or additive benefits (Khouma et al. 2025; Nuzhat et al. 2023).

An alternative strategy avoids the use of live microorganisms altogether by delivering their functional components directly. Postbiotics, defined as inanimate microorganisms and/or their bioactive components that confer health benefits, offer potential advantages in terms of safety and implementation, particularly in LMIC settings. These include reduced reliance on cold‐chain logistics and a lower theoretical risk of microbial translocation in vulnerable populations. The consensus definition proposed by the International Scientific Association for Probiotics and Prebiotics (ISAPP) has been widely adopted and provides a standardized framework for the classification and evaluation of postbiotic interventions in clinical and translational studies (Salminen et al. 2021; Vinderola et al. 2024).

#### 3.4.2 Evidence From Clinical and Preclinical Studies on Growth Outcomes

Animal models provide strong proof‐of‐concept that specific probiotic strains can restore gut microbiota and alleviate systemic inflammation. Despite this theoretical promise, empirical data from human RCTs is highly variable. Systematic reviews of clinical interventions in low‐resource settings suggest that host‐related factors, such as severe EED and poor WASH conditions, often blunt the efficacy seen in controlled preclinical models. Among children aged 0–59 months in LMICs, the certainty of evidence remains low, with pooled analyses suggesting potential benefits for ponderal growth (e.g., weight gain), whereas effects on linear growth are smaller and inconsistent across intervention types (Khouma et al. 2025). Similarly, a 2024 meta‐analysis of 12 RCTs (*n* ≈ 3086 malnourished children) reported modest increases in body weight and small, statistically variable gains in height following probiotic or synbiotic supplementation, with confidence constrained by substantial heterogeneity in microbial strains, dosing regimens, intervention duration, and study design (Paiandeh et al. 2024).

Evidence from individual trials provides mechanistic context for these aggregate findings. In young infants with severe acute malnutrition, a randomized trial in Bangladesh demonstrated higher rates of weight gain following supplementation with *Bifidobacterium infantis* EVC001 or a synbiotic formulation combining EVC001 with lacto‐N‐neotetraose (LNnT), with more pronounced effects on ponderal than linear growth over short follow‐up. These results support the concept that microbiome‐targeted interventions may enhance short‐term nutrient utilization during nutritional rehabilitation, whereas improvements in linear growth likely require longer intervention duration and more complete resolution of EED (Nuzhat et al. 2023).

In stunting contexts, attribution of growth effects is further complicated by multi‐component interventions. An Indonesian randomized placebo‐controlled trial evaluating *Lactiplantibacillus plantarum* IS‐10506 alongside a UHT‐treated postbiotic comparator in stunted children receiving functional food supplementation and WASH support reported growth improvements but also highlighted the difficulty of disentangling the specific contribution of the biotic intervention from concurrent co‐interventions, an important methodological challenge for future trials (Surono et al. 2025).

A clearer benchmark for growth‐relevant microbiome modulation is provided by microbiota‐directed foods. A 2024 EBioMedicine study of a microbiota‐directed complementary food (MDCF) in Bangladeshi children demonstrated sustained improvements in linear growth alongside age‐appropriate microbiome maturation and functional metabolic shifts. Unlike conventional biotic supplementation, the MDCF strategy was explicitly designed to correct microbiome immaturity and gut dysfunction, thereby strengthening the biological plausibility of microbiome‐targeted nutraceutical approaches, including synbiotic and postbiotic strategies, when interventions are sufficiently comprehensive to restore gut function and attenuate EED‐associated inflammation (Mostafa, Hibberd, et al. 2024; Mostafa, Sthity, et al. 2024).

Taken together, these findings highlight several practical considerations for nutraceutical development in stunting. Clinical effectiveness depends on clear strain and substrate specificity, as the term “probiotic” alone does not define a mechanism. Growth outcomes are shaped by the selected strain, dose, viability, and delivery format (Khouma et al. 2025; Paiandeh et al. 2024). In addition, sustained improvements in linear growth are more likely to require longer intervention periods and direct targeting of EED. Short‐duration trials tend to affect ponderal growth rather than height outcomes, indicating that meaningful gains in HAZ often depend on integration with adequate protein intake, sufficient micronutrient status, and supportive water, sanitation, and hygiene interventions (Khouma et al. 2025; Otiti et al. 2025).

Finally, while postbiotics offer practical advantages in low‐resource settings, they require strict ISAPP‐aligned validation (Salminen et al. 2021; Vinderola et al. 2024). Overall, despite their mechanistic promise against EED, clinical realities limit biotic therapies. Synbiotic adjuncts to macronutrients (e.g., LNS) successfully improve ponderal growth and gut barriers; however, standalone biotics rarely improve linear growth (HAZ/LAZ), as poor WASH conditions and chronic infections often overwhelm their effects. Large‐scale RCTs remain essential to establish optimal strains, dosing regimens, and postbiotic efficacy for rescuing stunted growth.

#### 3.4.3 Prospects and Trial Design Recommendations

**FIGURE 5:**
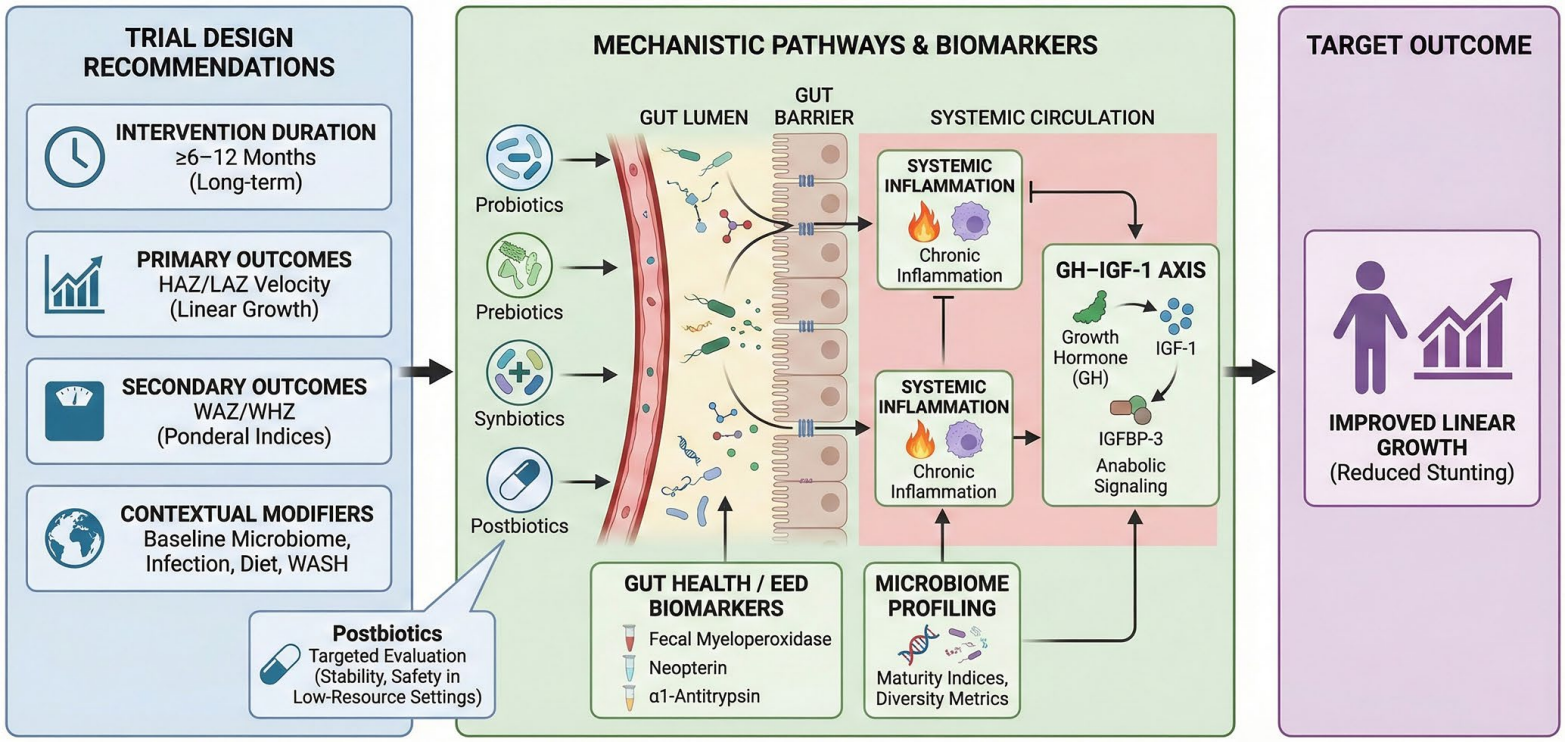
Framework for future clinical trials of microbiome‐targeted nutraceuticals in stunting, illustrating proposed trial design and mechanistic pathways linking probiotics, prebiotics, synbiotics, and postbiotics to linear growth outcomes. Long‐term interventions (≥ 6–12 months) prioritize HAZ/LAZ velocity as the primary endpoint, with WAZ/WHZ as secondary measures. Microbiome modulation is hypothesized to improve gut barrier function, reduce EED and systemic inflammation, and restore anabolic signaling via the GH–IGF‐1 axis, ultimately supporting linear growth. Microbiome profiling, EED biomarkers, and contextual modifiers (diet, infection burden, WASH) are incorporated to strengthen causal inference.

Within a proposed framework for future clinical trials of microbiome‐targeted nutraceuticals in stunting (Figure 5), several design considerations emerge as critical for demonstrating meaningful effects on linear growth. Trials evaluating probiotics, prebiotics, synbiotics, and postbiotics should employ longer intervention durations of at least 6 to 12 months to distinguish true linear growth responses from short‐term changes in body weight. Primary endpoints should prioritize HAZ/LAZ velocity, with ponderal indices such as weight‐for‐age or weight‐for‐height included as secondary outcomes to differentiate transient nutritional recovery from sustained linear catch‐up. To establish mechanistic links between microbiome modulation and growth outcomes, studies should incorporate validated markers of EED, including fecal myeloperoxidase, neopterin, and α1‐antitrypsin, together with systemic inflammatory indicators that reflect impaired gut barrier function and chronic inflammation known to interfere with GH–IGF‐1 signaling (Jannat et al. 2023; McGrath et al. 2017).

Microbiome profiling should extend beyond taxonomic descriptions to include microbiome maturity indices and diversity metrics, as age‐appropriate microbial maturation has been increasingly associated with growth trajectories in undernourished and stunted children (Sundjaya et al. 2024). Integration of endocrine readouts, particularly IGF‐1 and IGF‐binding protein‐3 (IGFBP‐3), is also warranted to assess whether improvements in gut health translate into restoration of systemic anabolic signaling, consistent with reported links between gut microbiota composition and IGF‐1 levels in pediatric populations (Rinanda et al. 2023).

To reduce heterogeneity and improve causal inference, trial designs should explicitly account for contextual modifiers, including baseline microbiome immaturity, infection burden, dietary quality (notably protein and micronutrient adequacy), and WASH conditions. Finally, postbiotic interventions, defined according to recent consensus as inanimate microbial preparations with demonstrated health benefits, warrant targeted evaluation for stability, safety, and growth‐related efficacy in low‐resource settings, where cold‐chain limitations and vulnerability to enteric infections pose significant implementation challenges (Hardjo and Selene 2024).

## 4 Prospects and Future Directions

### 4.1 Precision Nutrition for Stunting: Personalized Interventions Based on Genetics, Microbiome, and Epigenome

As summarized in Table 1, although various nutraceutical categories target distinct biological mechanisms of stunting, their clinical outcomes in pediatric populations remain highly heterogeneous. Unlike the clinical evidence discussed in previous sections, which relies on established interventions, the following methodologies represent forward‐looking, predictive frameworks that require robust future validation in stunted pediatric cohorts. Consequently, a key direction for future stunting interventions is a shift from uniform supplementation toward precision nutrition, in which the choice, dose, and duration of nutraceuticals are guided by individual biology and environmental context.

**TABLE 1:** Summary of nutraceutical categories, mechanisms, and clinical/preclinical outcomes in stunting.

Category	Specific intervention	Primary mechanism of action	Target population/study model	Key outcomes & observations
Macronutrients	LNS + Dairy (Milk protein/Whey)	Provides high‐density calories, essential amino acids to stimulate the GH–IGF‐1 axis and support muscle/bone accretion	Stunted children (e.g., 6–24 months) in LMICs (e.g., Uganda trial)	Modest improvements in length and weight velocities; dairy addition promotes anabolic recovery
Macronutrients	Omega‐3 PUFAs	Modulates lipid mediators, attenuates systemic inflammation, and supports cellular membrane integrity	Malnourished pediatric cohorts	Improved height SDS and weight gain in preliminary clinical intervention studies
Micronutrients	Micronutrient Powders (MNPs) & Zinc	Addresses critical enzymatic co‐factor deficits, reduces enteric mucosal permeability, and supports immune function	Infants and young children (e.g., Gaza Strip cohort, 6–30 months)	Significant reduction in the odds/prevalence of stunting; modest improvements in HAZ Z‐scores
Phytochemicals	Flavonoids, Curcumin, & Polyphenols	Exerts potent antioxidant and anti‐inflammatory effects; mitigates EED‐associated oxidative stress	Primarily Preclinical & in vitro models; mechanistically extrapolated to EED	Attenuates intestinal inflammation and modulates gut microbiota; clinical translation to human linear growth remains to be proven
Biotics & Microbiome	Probiotics (e.g., *Lactiplantibacillus plantarum* IS‐10506, *B. infantis* EVC001)	Restores gut barrier integrity, competitively excludes enteric pathogens, and produces immunomodulatory short‐chain fatty acids (SCFAs)	Stunted and malnourished infants/children (e.g., Indonesian and Bangladeshi trials)	Reductions in fecal inflammatory markers (e.g., calprotectin), improved gut maturation, and varied effects on weight/linear growth
Biotics & Microbiome	Prebiotics, Synbiotics, & Postbiotics	Provides substrate for beneficial taxa or delivers direct bioactive metabolites (e.g., cell wall fragments, bacteriocins)	Stunted pediatric cohorts and mechanistic models	Enhances intestinal epithelial barrier function without the risk of live bacterial translocation

A key direction for future stunting interventions is a shift from uniform supplementation toward precision nutrition, in which the choice, dose, and duration of nutraceuticals are guided by individual biology and environmental context. Differences in host genetics, gut microbiome composition, and epigenetic regulation influence nutrient metabolism, inflammatory responses, and growth efficiency, helping to explain why standardized interventions often produce variable growth outcomes (Elsayed and Saleh 2024).

From an implementation standpoint, microbiome‐based stratification is increasingly feasible. Children with microbiome immaturity or reduced functional capacity, such as limited short‐chain fatty acid production or altered bile acid metabolism, may benefit more from synbiotic, postbiotic, or microbiota‐directed food interventions. By contrast, children exposed to high enteric pathogen burdens or marked EED may require combined approaches that integrate microbiome modulation with infection control and WASH interventions to support linear growth. At the same time, growing evidence indicates that epigenetic markers can reflect early life nutritional and environmental stress and may help predict later growth trajectories through persistent effects on immune and metabolic gene regulation (Gkiouleka et al. 2025; Ramsteijn et al. 2024).

In the near term, research efforts should focus on the development of integrated risk and response profiles that combine microbiome maturity indices, EED biomarkers, and growth‐related endocrine markers such as IGF‐1 and IGF‐binding protein‐3. These profiles could inform adaptive trial designs that target biotic or postbiotic interventions to children most likely to benefit, while also identifying those for whom limited responsiveness points to the need for broader structural or environmental interventions. Ultimately, accelerating biomarker validation, expanding nutrigenomic profiling, and scaling adaptive trial designs for microbiota‐directed interventions remain the field's most urgent research priorities.

### 4.2 Systems Biology Approaches: Integrating Network Pharmacology, Bioinformatics, and Artificial Intelligence

Nutraceuticals seldom act through single molecular targets. Their effects typically extend across microbial metabolism, intestinal barrier function, immune regulation, and endocrine pathways involved in growth. Addressing this complexity requires systems biology approaches that move beyond reductionist models and support integrated analysis of interacting biological layers. Analytical pipelines that combine multi‐omics data, including metagenomics or metatranscriptomics, metabolomics, and proteomics, with host phenotypes such as EED biomarkers, inflammatory mediators, and IGF‐axis readouts can help identify mechanistic nodes that distinguish growth responders from non‐responders and guide the selection of translationally relevant targets.

Within this framework, network pharmacology provides a structured approach to linking nutraceutical components or postbiotic metabolites to host signaling pathways and biological processes. The growing use of AI‐based methods has further expanded the capacity to interrogate complex, high‐dimensional datasets, enabling pattern detection, target prioritization, and integration across heterogeneous studies. At the same time, recent methodological evaluations highlight vulnerabilities related to bias, data leakage, and overfitting, underscoring the need for transparent model development, robust reference datasets, and prospective validation (Cui et al. 2025; Wang et al. 2025; Yang et al. 2025; Zhang et al. 2023).

For stunting‐focused nutraceutical research, several priorities emerge. These include the adoption of standardized ontologies for interventions and outcomes, covering microbial strain identity, formulation and viability, EED biomarker panels, and growth velocity metrics. In parallel, analytical strategies should place greater emphasis on causal inference, particularly in linking microbiome and metabolic changes to recovery of GH–IGF‐1 axis signaling. Finally, the use of interpretable AI models, rather than purely predictive tools, is essential to ensure that computational findings yield biologically testable hypotheses that can inform experimental design and policy decisions.

### 4.3 Sustainable Nutraceutical Innovations: Local Resources, Biofortification, Community‐Based Strategies

For nutraceutical interventions to be scalable in LMICs, innovation must be grounded in practical considerations, including affordability, reliable supply chains, cultural acceptability, and environmental sustainability. Biofortification is among the most established population‐level approaches and can be achieved through conventional plant breeding, improved agronomic practices, and, where appropriate, biotechnological methods to increase the micronutrient content of staple foods. Recent syntheses support biofortification as a cost‐effective and scalable strategy to address micronutrient deficiencies, while also highlighting ongoing challenges related to seed distribution systems, consumer acceptance, nutrient retention during processing and cooking, and equitable access among vulnerable populations (Bouis et al. 2024; Naik et al. 2024). Furthermore, the formulation of next‐generation nutraceuticals must rigorously evaluate both synergistic and antagonistic interactions with conventional nutritional therapies, such as the known inhibition of iron absorption by certain polyphenols, to ensure they do not inadvertently compromise baseline micronutrient status (Gobinath et al. 2026; He and Chen 2024; Piskin et al. 2022).

Alongside biofortified staples, locally sourced nutraceuticals such as nutrient‐dense microgreens, legumes, seaweeds, and traditional fermented foods provide additional opportunities to improve dietary quality while reducing reliance on imported products. These approaches are particularly effective when integrated into community‐based delivery platforms, including women's groups, primary health care services, and “posyandu” (Indonesia's community‐based integrated health posts for maternal and child health), as well as school feeding programs and social protection schemes. To support sustained uptake, community‐centered strategies should incorporate context‐appropriate behavior change communication, basic quality assurance measures, and simple monitoring indicators, such as coverage, adherence, and adverse events. Co‐design with local stakeholders remains essential to ensure that nutraceutical innovations are acceptable and sustainable within the communities they are intended to serve.

### 4.4 Policy and Implementation Framework for LMICs

Translating microbiome‐targeted nutraceutical research into population‐level impact requires policy frameworks that embed these interventions within integrated stunting reduction strategies rather than treating them as stand‐alone products. Alignment with global nutrition targets and financing priorities is essential, together with delivery through established maternal and child health platforms and nutrition‐sensitive actions, including WASH, food security, education, and infection control. Global nutrition targets provide an accountability structure for these efforts, while recent investment frameworks have articulated the economic case for scaling interventions with demonstrated effectiveness.

Evidence from implementation science shows that success at scale depends not only on product efficacy but also on the strength of delivery systems. Key factors include workforce training, procurement and supply chains, and last‐mile distribution, as well as caregiver trust, community demand, and compatibility with local practices. Systematic assessments of micronutrient powder programs illustrate this interplay, identifying common barriers such as supply disruptions, poor adherence, and caregiver concerns, alongside facilitators including high‐quality counseling, community engagement, and reliable logistics (Sun et al. 2023).

Microbiome‐targeted nutraceuticals also raise specific policy considerations. Clear regulatory definitions and quality standards are needed, covering strain identification, claims related to viability and stability, and standardized characterization of postbiotic preparations. Crucially, robust safety monitoring in nutritionally vulnerable populations must be a policy priority, as the safety profiles, potential toxicity, and dosage limitations of novel nutraceuticals require rigorous evaluation. Children with stunting and EED possess altered metabolic and absorptive capacities, meaning standard pediatric dosing may be inappropriate. For instance, high‐dose iron supplementation in the presence of EED can inadvertently exacerbate oxidative stress and promote pathogenic enteric bacteria, while live biotherapeutics carry theoretical risks of translocation in severely immunocompromised or malnourished children. Furthermore, establishing Tolerable Upper Intake Levels (UL) for highly concentrated bioactives remains a critical knowledge gap. Current reviews indicate that regulatory approaches to postbiotics remain uneven across settings, underscoring the need for harmonized standards and transparent evidence requirements to support responsible scale‐up (Kim et al. 2026; Meena et al. 2025).

Finally, implementation in LMICs would benefit from stronger convergence governance, with shared indicators, coordinated budgeting across sectors, and accountability at district or local levels. Evaluations of Indonesia's multisectoral stunting strategy demonstrate how gaps in coordination and execution can weaken otherwise strong policy commitments, highlighting challenges that are directly relevant to scaling nutraceutical innovations within complex health and social systems (Rusdianti et al. 2025).

## 5 Conclusion

This review highlights the growing role of nutraceuticals as supportive approaches in the prevention and management of childhood stunting. In addition to conventional micronutrient supplementation, current evidence points to the importance of macronutrient quality, bioactive compounds, and microbiome‐targeted strategies in shaping intestinal function, inflammatory burden, and metabolic and endocrine pathways linked to impaired linear growth. Although reported effects on growth, particularly HAZ, are generally modest and variable, recent clinical evaluations illustrate how efficacy depends heavily on biological context. For instance, while adding targeted dairy protein to LNS yielded limited incremental growth benefits in populations with high EED burdens, targeted microbiota‐directed complementary foods (MDCFs) have demonstrated significant success in sustaining linear growth by correcting baseline microbiome immaturity. These findings emphasize the influence of biological context, intervention design, and treatment duration on clinical outcomes, as well as the need for caution when translating theoretical benefits into clinical practice.

While much of the current mechanistic understanding, particularly regarding the role of specific phytochemicals in modulating growth signaling pathways, is derived from preclinical and experimental models, advances in nutrigenomics, microbiome research, and epigenetics provide a strong rationale for more targeted nutritional strategies. Integrating these dimensions can help identify children who are more likely to respond to specific nutraceutical interventions and support the development of precision nutrition frameworks that address heterogeneity in growth responses. This approach is especially relevant in settings where EED, infection, and dietary inadequacy interact to limit growth potential.

Translation of these preclinical hypotheses and early nutraceutical research into meaningful reductions in stunting will depend on alignment with public health priorities and implementation systems in LMICs. Evidence‐based policy, well‐powered human pediatric clinical trials, regulatory clarity, and integration with broader interventions addressing diet quality, infection control, and WASH remain essential. Strengthening the connection between mechanistic understanding and real‐world application will be key to ensuring that nutraceutical strategies contribute to sustained improvements in child growth and health. Ultimately, establishing the biological efficacy of targeted nutraceuticals must be matched by an equal commitment to overcoming the logistical, regulatory, and cultural implementation barriers inherent to LMIC health systems.

## Author Contributions

**Chika Yamada:** formal analysis, investigation, writing – review and editing. **Trina Ekawati Tallei:** conceptualization, methodology, writing – original draft, writing – review and editing, visualization, supervision, project administration, funding acquisition, validation. **Souvia Rahimah:** conceptualization, methodology, formal analysis, investigation, data curation, visualization, writing – original draft, writing – review and editing, project administration, funding acquisition. **Youdiil Ophinni:** formal analysis, writing – review and editing, supervision. **Bonglee Kim:** writing – review and editing, supervision. **Hyo Jung Kim:** resources, writing – review and editing. **Min Choi:** resources, writing – review and editing. **Moon Nyeo Park:** resources, writing – review and editing. **Maghfirah Savitri:** methodology, formal analysis, investigation, data curation, writing – original draft, writing – review and editing.

## Funding

The publication charge was funded by Universitas Padjadjaran through the Indonesian Endowment Fund for Education (LPDP) on behalf of Indonesian Ministry of Higher Education, Science and Technology and manage under the EQUITY Program (Contract No. 4303/B3/DT.03.08/2025 and 3987/UN6.3.1/PT.00/2025).

## Conflicts of Interest

The authors declare no conflicts of interest.

## Data Availability

Data sharing not applicable to this article as no datasets were generated or analysed during the current study.
